# Antibacterial, Antifungal, and Insecticidal Potentials of *Oxalis corniculata* and Its Isolated Compounds

**DOI:** 10.1155/2015/842468

**Published:** 2015-03-19

**Authors:** Azizur Rehman, Ali Rehman, Ijaz Ahmad

**Affiliations:** Department of Chemistry, Kohat University of Science & Technology (KUST), Khyber Pakhtunkhwa, Kohat 26000, Pakistan

## Abstract

*Oxalis corniculata* is a common medicinal plant widely used against numerous infectious diseases. The agrochemical potential of methanolic extract, *n*-hexane, chloroform, ethyl acetate, and *n-butanol* fractions were assessed to measure the antibacterial, antifungal, and insecticidal activities of the plant. The crude, chloroform, and *n-butanol* soluble fractions showed excellent activities against *Escherichia coli*, *Shigella dysenteriae*, *Salmonella typhi*, and *Bacillus subtilis* but have no activity against *Staphylococcus aureus*. Similarly the crude, *n*-hexane, and chloroform fractions were also found to have significant activity against fungal strains including *Fusarium solani*, *Aspergillus flexneri*, and *Aspergillus flavus* and have no activity against *Aspergillus niger*. Chemical pesticides have shown very good results at the beginning, but with the passage of time the need was realized to use the natural plant sources for the safe control of insects. The current study will provide minor contribution towards it. High mortality rate was recorded for the crude extract and chloroform fraction against *Tribolium castaneum*. The two isolated compounds 5-hydroxy-6,7,8,4′-tetramethoxyflavone (**1**) and 5,7,4′-trihydroxy-6,8-dimethoxyflavone (**2**) were evaluated for antibacterial, antifungal, and insecticidal activities. The results showed that compound **2** was more active than compound **1** against the tested bacterial strains and insects.

## 1. Introduction

The progress in new antimicrobial agents to multidrug resistant pathogens for the treatment of various infectious diseases is of increasing interest. Therefore different plant extracts of many plants have been used locally against various pathogens, which showed positive results [1].

Higher plants and their extracts are used for the treatment of infectious disease as traditional medicines from the very beginning of life. About 80 percent of the world population defends upon the plant extracts [2]. But there are only a small percentage of these plants that have been subjected to pharmacological or biological screening [3]. Microbes which are free to move around the world cause different infections in human races. In developing countries about 43 percent of total deaths are due to infectious diseases. WHO claims significant control in major infectious diseases [4]. Due to jumbled use of existing antimicrobial drugs pathogenic bacteria have developed resistance against wide range of antibiotics. The *β*-lactamase producers like* Staphylococcus aureus*,* Escherichia coli*,* Klebsiella pneumonia*, and many others have become a major therapeutic problem, resulting in increased treatment failure and health care cost. Microbiologists from all over the world are in search to formulate new antimicrobial drugs and evaluate the efficiency of plant products to replace chemical antimicrobial agents [5]. Medicinal plants extracts have shown to serve as a cheap source of antimicrobial agents against pathogenic microbes. These extracts have biologically active compounds, which make them attractive fowler for preying these pathogens [6].

Annual postharvest losses are resulting due to insect damage, microbial deterioration, and many other environmental factors such as humidity, aeration, temperature, and cleanliness of the massive storage and are expected to be 10–25% of production throughout the world. Insects are the major problem in stored grain because they destroy the quantity and quality of the grains [7]. Higher plants and their extracts can be used for environmentally safe control of insects. It is claimed that 99 families, 276 genera, and 346 species of medicinal plants have environment- friendly insecticidal properties [8]. Plant extracts or pure compounds control insects in several ways, including antifeedant, growth inhibitors, toxicity, mortality suppression of reproductive behavior, and fertility [9]. Synthetic chemical pesticides have some serious flaws to environmental and health related concerns. It is expected that only in America about 200 people are dying every year due to this pesticidal poisoning [10]. These problems resulted in renewed interest in formulating new botanical pesticides, which could be nonhazardous, effective, biodegradable, and of low cost and pose less threat to the environment [11].

The aim of the present study was to evaluate the antibacterial, antifungal, and insecticidal activities of different fractions of* Oxalis corniculata*. These include methanol,* n*-hexane, chloroform, ethyl acetate, and* n-*butanol soluble fractions. Selection of* Oxalis corniculata* fractions was based on the fact that these fractions were not previously screened for these activities.

## 2. Material and Methods

### 2.1. Collection of Plant Sample

*Oxalis corniculata* was collected from District Buner, KPK, Pakistan in June 2012. Plant was identified by plant taxonomist Dr. Nisar Ahmad, Chairman Department of Botany, Kohat University of Science & Technology, Kohat, KPK, Pakistan, and a voucher specimen number 169 was stored there in the herbarium.

### 2.2. Preparation of Extract

Plant was shade dried at room temperature and 1 kg dried plant materials were soaked in methanol to obtain methanolic extract. Extract was evaporated under reduced pressure to dryness; the residue was weighed (81 g) and redissolved in distilled water. The aqueous solution of the plant extract was subjected to different solvents on the basis of increasing polarity like* n*-hexane, chloroform, ethyl acetate, and* n-*butanol to get the respective* n*-hexane, chloroform, ethyl acetate, and* n-*butanol solvent soluble fractions with the ratio of 15 g (*n*-hexane fraction), 22 g (chloroform fraction), 16 g (ethyl acetate fraction), 14 g (*n*-butanol fractions), and 10 g (aqueous fraction), respectively. All the fractions were dried at low pressure using rotary evaporator and stored at 4°C.

### 2.3. Microbial Strains

To evaluate the antimicrobial activity of different fractions of* Oxalis corniculata*, nine microbial species were used, five of which were bacterial pathogens including* Bacillus subtilis* (ATCC7966),* Staphylococcus aureus* (ATCC 12600),* Escherichia coli* (ATCC8677),* Shigella dysenteriae* (ATCC29027), and* Salmonella typhi* (ATCC0650) and four fungal species including* Fusarium solani*,* Aspergillus flexneri*,* Aspergillus flavus*, and* Aspergillus niger* were taken from culture collection of Department of Microbiology, Kohat University of Science & Technology, Kohat, Pakistan.

## 3. Antibacterial Assay

### 3.1. Preparation of Bacterial Inoculums

The bacterial strains were subcultured to get fresh cultures of bacteria. For this purpose a single colony from bacterial strain was inoculated on nutrient broth. The broth was incubated for 24 hours at 37°C. 14 g of nutrient agar media was dissolved in 1 L of distilled water at PH 7 and autoclaved for 20 minutes at 121°C. The media were allowed to cool down to 45°C and poured to petri plates (14 cm) for preparing 75 mL of solid media. Using sterile cork borer (8 mm) 7 wells per plate were made in the solidified media. Agar diffusion method was used for antibacterial activity [12]. Bacterial culture was inoculated on the surface of solid media. The crude extract and fractions were dissolved in dimethyl sulfoxide (DMSO) at the same concentration of 2 mg/mL to prepare stock solutions. From the stock solutions, 100 *μ*L was poured into respective wells. Cefixime was used as positive control while DMSO was used as a negative control. The zones of inhibition of crude extract and fractions were measured in mm after 24 hours of incubation at 37°C and compared with the zone of inhibition of standard drug Cefixime [12].

## 4. Antifungal Assay

### 4.1. Preparation of Fungal Inoculums

Four fungal strains including* Aspergillus flexneri*,* Aspergillus niger*,* Aspergillus flavus*, and* Fusarium solani* were tested for the antifungal activity. Fungal strains were subcultured in potato dextrose agar (PDA) and incubated for 7 days at 28°C. To evaluate the antifungal activity the disk diffusion method was used [3]. Fungal strains were inoculated on the potato dextrose agar plate (PDA) by point inoculation. 100 *μ*L of solution (2 mg/mL in DMSO), pure DMSO (−ve control), and antibiotic terbinafin 2 mg/mL (+ve control) were used. After incubation of 7 days at 28°C, the fungal activities were noted.

## 5. Insecticidal Assay

The insects* Ephestia cautella* and* Tribolium castaneum* were taken from Department of Zoology, Kohat University of Science & Technology, Kohat, Kpk, Pakistan. The insecticidal activity was determined by direct contact application [13]. 60 mm of petri dish was used to conduct the surface film activity of all the extract by dissolving 50 mg/mL of crud extract and fractions in DMSO. Extracts were sprayed on to the lower part of the petri dish and allowed to dry out. The insects were released in these treated petri dishes. Pure DMSO was taken as a standard. These treated petri dishes having insects were kept at room temperature in a secured place. The result was observed from time to time starting from 30 minutes to 48 hours and finally recorded. The mortality of insects was confirmed by using simple microscope to check any movement of their organs. In last, the living (if any) insects were recovered and submitted to their respective department [14].

The percentage mortality rate was determined by the following formula:

(1) Mortality% =100−number  of  survival  insamplesnumber  of  survival  in  control×100.

## 6. Results and Discussion

Medicinal plants are the most prominent source of natural products against various common infectious microbes. The appearance of multidrug-resistant infectious microbes, high cost of synthetic compounds, and uninvited side effects of certain drugs insisted the new era to search for the new curative agents from alternative and low cost sources like medicinal plants.

The current attempt was made due to increasing resistance of different pathogens (bacteria and fungi) and insects against available antibiotics and insecticides, respectively. Plants extracts and compounds are of innovative interest as safe and health friendly antimicrobial and insecticidal agents. As a result the different fractions of* Oxalis corniculata* were screened for their antimicrobial and insecticidal activities.

To assess the antimicrobial activity of* Oxalis corniculata*, the microbial strains used were tested bacterial pathogens including* Escherichia coli*,* Bacillus subtilis*,* Staphylococcus aureus*,* Salmonella typhi*, and* Shigella dysenteriae*; the fungal species including* Fusarium solani, Aspergillus flexneri*,* Aspergillus flavus*, and* Aspergillus niger* were used. For the evaluation of insecticidal activity the insects used were* Ephestia cautella* and* Tribolium castaneum*. The plant solvent soluble extracts showed a broad spectrum of activities against used microbes and insects. The crude extract,* n-butanol*, and ethyl acetate soluble fractions showed excellent activities against* Escherichia coli*,* Salmonella typhi*, and* Bacillus subtilis*. Similarly crude extract,* n*-hexane, chloroform, and* n-butanol* soluble fractions were active against* Shigella dysenteriae* but not active in case of ethyl acetate soluble fraction. No activity was recorded against* Staphylococcus aureus* as shown in Table 1. Similarly the crude extract,* n*-hexane, and soluble fractions were also found to have significant activity against fungal strains including* Fusarium solani* and* Aspergillus flexneri*; the crude extract, chloroform, and ethyl acetate soluble fractions were active against* Aspergillus flavus*. The* n-butanol* soluble fraction was only active against* Aspergillus flexneri*; also chloroform, ethyl acetate, and* n-butanol* soluble fractions were inactive against* Fusarium solani*. In case of* Aspergillus niger* all the fractions showed no activity as shown in Table 2. Taley et al., 2012 [15], investigated the methanol and aqueous extracts of* O. corniculata* leaves for antibacterial activities against 5 bacterial strains:* E. coli*,* S. aureus*,* P. aeruginosa*,* P. vulgaris*, and* B. subtilis*. They recorded zone of inhibition in the range of 6–14 mm against these pathogens among which* B. subtilis* showed maximum zone of inhibition (14 mm), while, in our present investigation, the whole plant (*O. corniculata*) crude extract, its solvent soluble fractions, and the isolated compounds were evaluated for antibacterial and antifungal activities and obtained significant results.

The maximum toxicity of plant against* Ephestia cautella* and* Tribolium castaneum* was recorded for the crud extract and chloroform soluble fraction with highest mortality rate especially for the* Tribolium castaneum* (up to 62%).* n-*Butanol soluble fraction also showed valuable mortality (46%) in 48 hours as shown in Table 3. The lowest mortality rate was recorded for ethyl acetate and* n-butanol* soluble fractions against* Ephestia cautella* (08%) after 48 hours; however it was nonactive till 24 hours. No significant change in toxicity was observed in the experiment with passage of time. The comparative study shows that the plant is more toxic against* Tribolium castaneum* than* Ephestia cautella*. There is no literature available on the insecticidal activity of Oxalis species to compare with the present results.

### 6.1. Compounds Isolated from* Oxalis corniculata*

**Figure 1:**
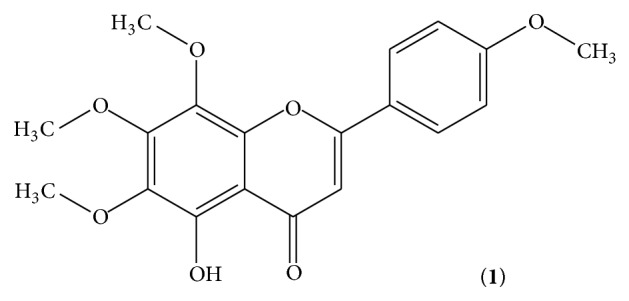
5-Hydroxy-6,7,8,4′-tetramethoxyflavone.

**Figure 2:**
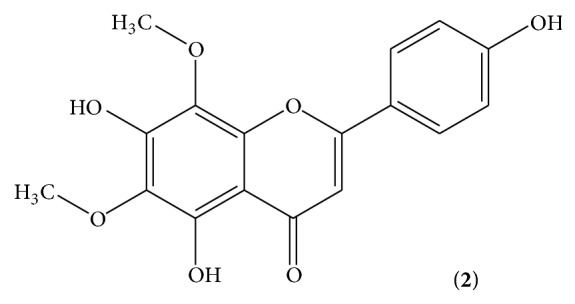
5,7,4′-Trihydroxy-6,8-dimethoxyflavone.

The chloroform soluble fraction was subjected to column chromatography and as a result two known compounds, compound** 1** (Figure 1) and compound** 2** (Figure 2), were isolated whose structures were elucidated with the help of EI, HREIMS, 1D, and 2D NMR techniques. The two isolated compounds** 1** and** 2** were subjected to antibacterial, antifungal, and insecticidal activities. The result showed that compound** 2** was more active than compound** 1** against the tested organisms. Antibacterial activity of compound** 2** was potent as compared to compound** 1**. Both the compounds were active against all the tested bacteria except* S. aureus* as shown in Table 4. While compounds** 1** and** 2** showed negative activity against* Fusarium solani*,* Aspergillus flavus*,* Aspergillus niger*, and* Aspergillus flexneri* which indicates that these compounds are inactive against these species of fungi.  Table 5 shows that insecticidal activity of compound** 2** was also potent as compared to that of compound** 1**. Both the compounds showed a good mortality rate against the* Tribolium castaneum*.

## 7. Conclusions

The present study suggests that* Oxalis corniculata* has good antibacterial, antifungal, and insecticidal properties and can be used for the treatment of infections and control of insects. The plant extracts could be a new source for antibiotics and pesticides with minimum noxious effects on the environment. Further studies may also lead to isolate and characterize the active compounds of the plant extracts and to elucidate their biological mechanisms of action.

## Figures and Tables

**Table 1:** Showing zones of inhibition (mm) against different bacteria.

S. number	Fractions	Gram positive bacteria		Gram negative bacteria		
		*B. subtilis *	*S. aureus *	*E. coli *	*S. dysenteriae *	*S. typhi *
1	Crude	24	00	21.3	17	11
2	*n*-Hexane	14	00	00	14.6	09
3	Chloroform	11.3	00	11	12	00
4	Et. acetate	14.8	00	11.7	00	8.4
5	*n-Butanol *	24	00	16.5	13.2	08

**Table 2:** Showing activity of different fractions against fungi.

S. number	Fractions	*A. flavus *	*A. flexneri *	*A. niger *	*F. solani *
1	Crude	+	+	−	+
2	*n*-Hexane	−	+	−	+
3	Chloroform	+	+	−	−
4	Et. acetate	+	−	−	−
5	*n-Butanol *	−	+	−	−

**Table 3:** Showing percentage mortality rate of insects against different fractions of *Oxalis corniculata*.

S. number	Fractions	*T. Castaneum*	*T. castaneum*	*E. cautella*	*E. cautella*
		*24 hours *	*48 hours *	*24 hours *	*48 hours *
1	Crude	53	57	34	49
2	*n*-Hexane	15	23	22	25
3	Chloroform	62	62	44	47
4	Et. acetate	11	14	00	08
5	*n-Butanol *	38	46	00	08

**Table 4:** Zone of inhibition (mm) of compounds **1** and **2** against different bacteria.

S. number	Compounds	*E. coli *	*S. dysenteriae *	*S. aureus*	*S. typhi *	*B. subtilis *
1	Compound **1**	13.3	14.9	00	10.1	14.3
2	Compound **2**	16.5	14.6	00	11.7	15.6

**Table 5:** Percentage mortality rate of *T. castaneum* against compounds **1** and **2**.

S. number	Compounds	*T. castaneum *	*T. castaneum *
		*24 hours *	*48 hours *
1	Compound **1**	34.21%	38.47%
2	Compound **2**	41.56%	45.33%

## References

[1] Obeidat M. (2011). Antimicrobial activity of some medicinal plants against multidrug resistant skin pathogens. *Journal of Medicinal Plants Research*.

[2] Rahman M. S., Khan M. M. H., Jamal M. A. H. M. (2010). Anti-bacterial evaluation and minimum inhibitory concentration analysis of *Oxalis corniculata* and *Ocimum santum* against bacterial pathogens. *Biotechnology*.

[3] Mahesh B., Satish S. (2008). Antimicrobial activity of some important medicinal plant against plant and human pathogens. *World Journal of Agricultural Sciences*.

[4] Hussain F., Ahmad B., Hameed I., Dastagir G., Sanaullah P., Azam S. (2010). Antibacterial, antifungal and insecticidal activities of some selected medicinal plants of polygonaceae. *African Journal of Biotechnology*.

[5] Maji S., Dandapat P., Oja D. (2010). In vitro anti microbial potentialities of different solvent extracts of ethnomedicinal plants against clinically isolated human pathogens. *Journal of Phytology*.

[6] Khan R. A., Khan M. R., Sahreen S., Bokhari J. (2010). Antimicrobial and phytotoxic screening of various fractions of *Sonchus asper*. *African Journal of Biotechnology*.

[7] Haouas D., Kamel M. B., Hamouda M. H. B. (2008). Insecticidal activity of flower and leaf extracts from *Chrysanthemum* species against *Tribolium confusum*. *Journal of Plant Protection*.

[8] Singh R. K., Dhiman R. C., Mittal P. K. (2006). Mosquito larvicidal properties of *Momordica charantia* Linn (Family: Cucurbitaceae). *Journal of Vector Borne Diseases*.

[9] Jbilou R., Ennabili A., Sayah F. (2006). Insecticidal activity of four medicinal plant extracts against *Tribolium castaneum* (Herbst) (Coleoptera: Tenebrionidae). *African Journal of Biotechnology*.

[10] Sharm P. P., Pardeshi A. B., Vijigiri D. (2011). Bioactivity of some medicinal plant extracts against *Musca domestica* L.. *Journal of Ecobiotechnology*.

[11] Khan T., Ahmad M., Khan R., Khan H., Choudhary M. I. (2008). Phytotoxic and insecticidal activities of medicinal plants of Pakistan, *Trichodesma indicum*, *Aconitum laeve* and *Sauromatum guttatum*. *Journal of the Chemical Society of Pakistan*.

[12] Khan A. M., Qureshi R. A., Gillani S. A., Ullah F. (2011). Antimicrobial activity of selected medicinal plants of Margalla hills, Islamabad, Pakistan. *Journal of Medicinal Plant Research*.

[13] Azizuddin, Choudhary M. I. (2011). Antibacterial, phytotoxic, insecticidal and cytotoxic potential of *Vitex agnus-castus*. *Journal of Medicinal Plant Research*.

[14] Islam M. S., Zahan R., Alam M. B. (2011). Studies on antibacterial and insecticidal activities of *Suregada multiflora*. *Libyan Agriculture Research Center Journal International*.

[15] Taley S. L., Pethe A. S., Rothe S. P. (2012). Studies on antibacterial activity of some plant extracts. *International Multidisciplinary Research Journal*.

